# Induction of osteoarthritis by injecting monosodium iodoacetate into the patellofemoral joint of an experimental rat model

**DOI:** 10.1371/journal.pone.0196625

**Published:** 2018-04-26

**Authors:** Ikufumi Takahashi, Taro Matsuzaki, Hiroshi Kuroki, Masahiro Hoso

**Affiliations:** 1 Section of Rehabilitation, Kanazawa University Hospital, Ishikawa, Japan; 2 Department of Motor Function Analysis, Human Health Sciences, Graduate School of Medicine, Kyoto University, Kyoto, Japan; 3 Division of Health Sciences, Graduate School of Medical Science, Kanazawa University, Ishikawa, Japan; SERGAS and IDIS, SPAIN

## Abstract

This study aimed to investigate the histopathological changes in the patellofemoral joint using a rat model of osteoarthritis that was induced using monosodium iodoacetate, and to establish a novel model of patellofemoral osteoarthritis in a rat model using histopathological analysis. Sixty male rats were used. Osteoarthritis was induced through a single intra-articular injection of monosodium iodoacetate in both knee joints. Animals were equally divided into two experimental groups based on the monosodium iodoacetate dose: 0.2 mg and 1.0 mg. Histopathological changes in the articular cartilage of the patellofemoral joint and the infrapatellar fat pad were examined at 3 days, 1 week, 2 weeks, 4 weeks, 8 weeks, and 12 weeks after the monosodium iodoacetate injection. In the 1.0-mg group, the representative histopathological findings of osteoarthritis were observed in the articular cartilage of the patellofemoral joint over time. Additionally, the Osteoarthritis Research Society International scores of the patellofemoral joint increased over time. The synovitis scores of the infrapatellar fat pad in both groups were highest at 3 days, and then the values decreased over time. The fibrosis score of the infrapatellar fat pad in the 1.0-mg group increased with time, whereas the fibrosis score in the 0.2-mg group remained low. Representative histopathological findings of osteoarthritis were observed in the articular cartilage of the patellofemoral joint in a rat model of osteoarthritis induced using monosodium iodoacetate. With appropriate selection, this model may be regarded as an ideal patellofemoral osteoarthritis model.

## Introduction

The most common condition that affects the knee joints is osteoarthritis (OA) [1–3]. The knee joint consists of two compartments: the tibiofemoral (TF) compartment and the patellofemoral (PF) compartment [4]. OA in the knee can occur solely in the TF joint or in the PF joint, or it can be present in both joints [3–5]. Most research on OA has focused on the TF joint, although the prevalence of isolated OA in the PF joint (PFOA) might be higher than that of isolated OA in the TF joint (TFOA) [6–9]. Of the patients with knee pain, approximately 10 to 24% have isolated PFOA [10, 11]. Collins et al. reported that the radiographic signs of PFOA are associated with symptoms such as pain and disability [12]. There is evidence that crepitation, anterior knee pain, and difficulty during stair ambulation are more frequently associated with PFOA than with TFOA [2]. Despite emerging evidence regarding the important role that the PF joint plays in knee OA and despite the substantial individual and societal burden of PFOA, there is surprisingly little evidence of effective treatments and conservative and surgical management [3, 12]. We considered that one of the reasons for this problem is the lack of an established animal model for PFOA.

Animal models for OA are well established, and they serve as an important adjunct and surrogate for studies of OA in humans [13, 14]. These models provide a means to studying the pathophysiology of OA, and aid in the development of therapeutic agents and biological markers for diagnosing and prognosing disease [13]. Animal models of OA belong to three general categories of in vivo OA models: 1) naturally occurring OA models, including genetically modified animals; 2) models of the initiation or acceleration of joint degeneration developed following surgery or other trauma; and 3) models developed through intra-articular injection of chondrotoxic or proinflammatory substances [15]. However, these models and most studies using established animal models have focused on TFOA. Therefore, animal models of PFOA have not been established, resulting in the lack of evidence regarding PFOA in humans, as well as the histology, pathology, mechanisms, and effective treatments for PFOA.

In rats, the monosodium iodoacetate (MIA) model is well established, and the induced OA resembles human degenerative OA in terms of the histological and pain-related behaviors [16–19]. MIA is a metabolic inhibitor that breaks down the cellular aerobic glycolysis pathway, and consequently, induces cell death by inhibiting the activity of glyceraldehyde-3-phosphate dehydrogenase in chondrocytes [20]. Intra-articular injection of MIA leads to a reduction in the number of chondrocytes and subsequent histological and morphological articular alterations, which are similar to the changes in human OA [16–19]. In addition to the histopathological changes in the articular cartilage, intra-articular injection of MIA induces histopathological changes in the synovial membrane. Udo et al. reported that the thickness of the cell lining at the surface of the infrapatellar fat pad (IFP) increased, and the IFP inflammation scores revealed that 0.2 mg of MIA induced reversible synovitis, whereas 1.0 mg of MIA induced fibrosis of the IFP body [21]. We previously reported that the injection of MIA induced histopathological changes of the articular cartilage, as well as of the synovial membranes and joint capsules [14]. On the basis of these reports and findings, we supposed that intra-articular injection of MIA may induce histopathological changes in all the tissues within the knee joint, and that an injection of MIA may induce histopathological changes similar to PFOA.

Therefore, this study aimed to investigate the histopathological changes in the PF joint using a rat model of OA that was induced by MIA, and to establish a novel rat model of PFOA using histopathological analysis.

## Materials and methods

### Experimental animals and animal care

This protocol was approved by the Animal Research Committee of the Kanazawa University Graduate School of Medicine in Kanazawa, Japan (approval no. 173831), and conducted in accordance with the ARRIVE guidelines [22] and the guidelines for the care and use of laboratory animals at Kanazawa University.

Sixty-five male Wistar rats (9 weeks old; mean mass, 274.5 ± 7.8 g) were used for this study. Five rats were used as the control group, and 60 rats were used as the experimental group. The animals were housed under normal conditions for 1 week before the start of the experiments to acclimate them to the environment. One to two rats were housed per cage in a sanitary, ventilated room with controlled temperature, humidity, and a 12-hour/12-hour light-dark cycle; food and water were provided ad libitum.

### Induction of the osteoarthritis model

OA was induced through a single intra-articular injection of MIA, as previously described [14, 18, 19, 23]. MIA (cat. #I2512; Sigma, St. Louis, MO, USA) was dissolved in 30 μL of sterile saline, and the dose of MIA was 0.2 mg or 1.0 mg. Under anesthesia with isoflurane inhalation, both knees were shaved and disinfected. Then, an incision was made at the center of the knee to expose the patellar ligament. Each rat was positioned on its back, and the leg was flexed 90° at the knee joint. The patellar ligament was palpated below the patella, and MIA was injected into the medial side of the ligament of both knees using a 29-gauge, 0.5-inch needle. Care was taken to ensure that the needle was not advanced too far into the cruciate ligaments. Two groups were created according to the dose of MIA (0.2 mg and 1.0 mg; n = 30 per group). Later, 30 of the 60 rats were randomly allocated to one of six subgroups that were evaluated at 3 days, 1 week, 2 weeks, 4 weeks, 8 weeks, and 12 weeks postoperatively (n = 5 per subgroup). After the MIA injection and during the experimental period, no intervention was performed in any of the animals. Additionally, no analgesics or anti-inflammatory drugs were used.

### Histological preparation

Immediately after the animals were sacrificed by an intraperitoneal overdose of pentobarbital sodium (80 μg/g body weight), both hindlimbs were disarticulated at the hip joint, and all knees were fixed in 10% neutral-buffered formalin for 72 hours and decalcified using Decalcifying Solution A (Plank-Rychlo Solution, 7% w/v AlCl_3_6H_2_O, 5% formic acid, and 8.5% HCl; Wako Pure Chemical Industries, Ltd., Osaka, Japan) for 72 hours. The right and left knees were excised sagittally and frontally, respectively, to evaluate the histopathological changes in the PF and TF joints. Specimens were deacidified in 5% sodium sulfate solution for 72 hours, dehydrated in 100% ethanol after washing with water, and embedded in paraffin wax. Decalcified paraffin specimens in the sagittal and frontal planes were prepared according to the Osteoarthritis Research Society International (OARSI) recommendations described by Gerwin et al. [24]. The 3-μm sectioned slides were stained separately with hematoxylin and eosin (HE) and 0.1% safranin-O fast green for 5 minutes [25]. Then, the slides were sequentially dehydrated in 70%, 80%, 90%, and 100% ethanol. Finally, sections were cleared in xylene. A light microscope and a digital camera (BX-51 and DP-50; Olympus Corporation, Tokyo, Japan) were used to capture and evaluate the histopathological features of the articular cartilage and IFP.

### Determination of regions for assessment

**Fig 1:**
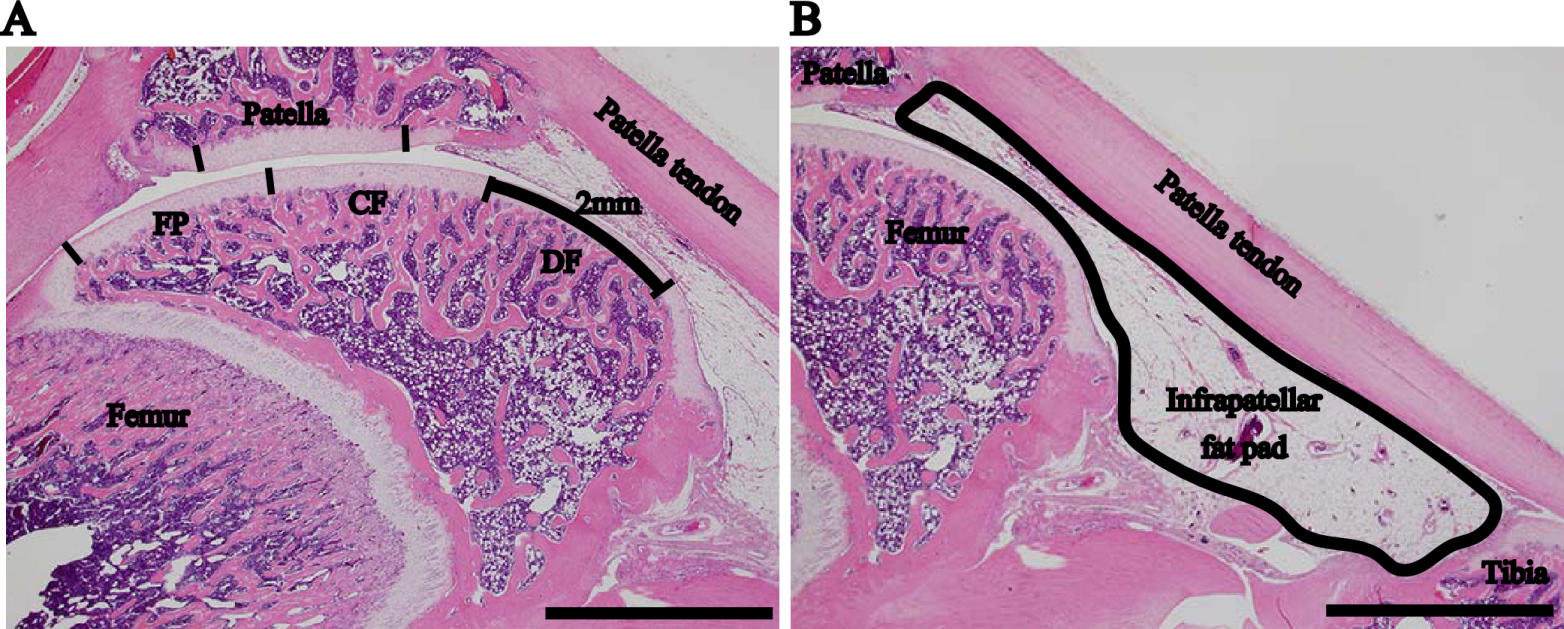
Histopathological assessment of regions in the sagittal section at the center of the patellofemoral joint. (A) Assessment of the articular cartilage with hematoxylin and eosin staining was performed at each region (2-mm wide). (B) Infrapatellar fat pad. Scale bar = 2 mm. Articular cartilage region: proximal femur (FP), central femur (CF), distal femur (DF), and patella.

To histopathologically analyze the PF joint, the right knees were excised sagittally, and the paraffin specimens were sliced at the center of the patellar groove to observe the cruciate ligaments at the center of the medial and lateral condyles. We determined the regions of the articular cartilage and IFP for the assessment of the PF joint according to the modified method described by Nomura et al. [26] (Fig 1). The proximal femur (FP) was defined as the proximal region of the articular cartilage located in the patellar groove. The distal femur (DF) was defined as the distal region of the articular cartilage located in the patellar groove. The central femur (CF) was situated between the FP and DF. The patella was evaluated at the central regions. A histological assessment was performed for each 2-mm-wide region. To evaluate the degrees of synovitis and fibrosis, the surface of the synovial membrane and the body of the IFP were determined as the assessment regions (Fig 1).

For histopathological analysis of the TF joint, the left knees were excised frontally, and the paraffin specimens were sliced at the center of the medial TF joint. To evaluate the degree of osteoarthritic development, the articular cartilage of the femur and tibia at the medial portion of the knee joint was determined as the assessment region.

### Histological analysis

**Fig 2:**
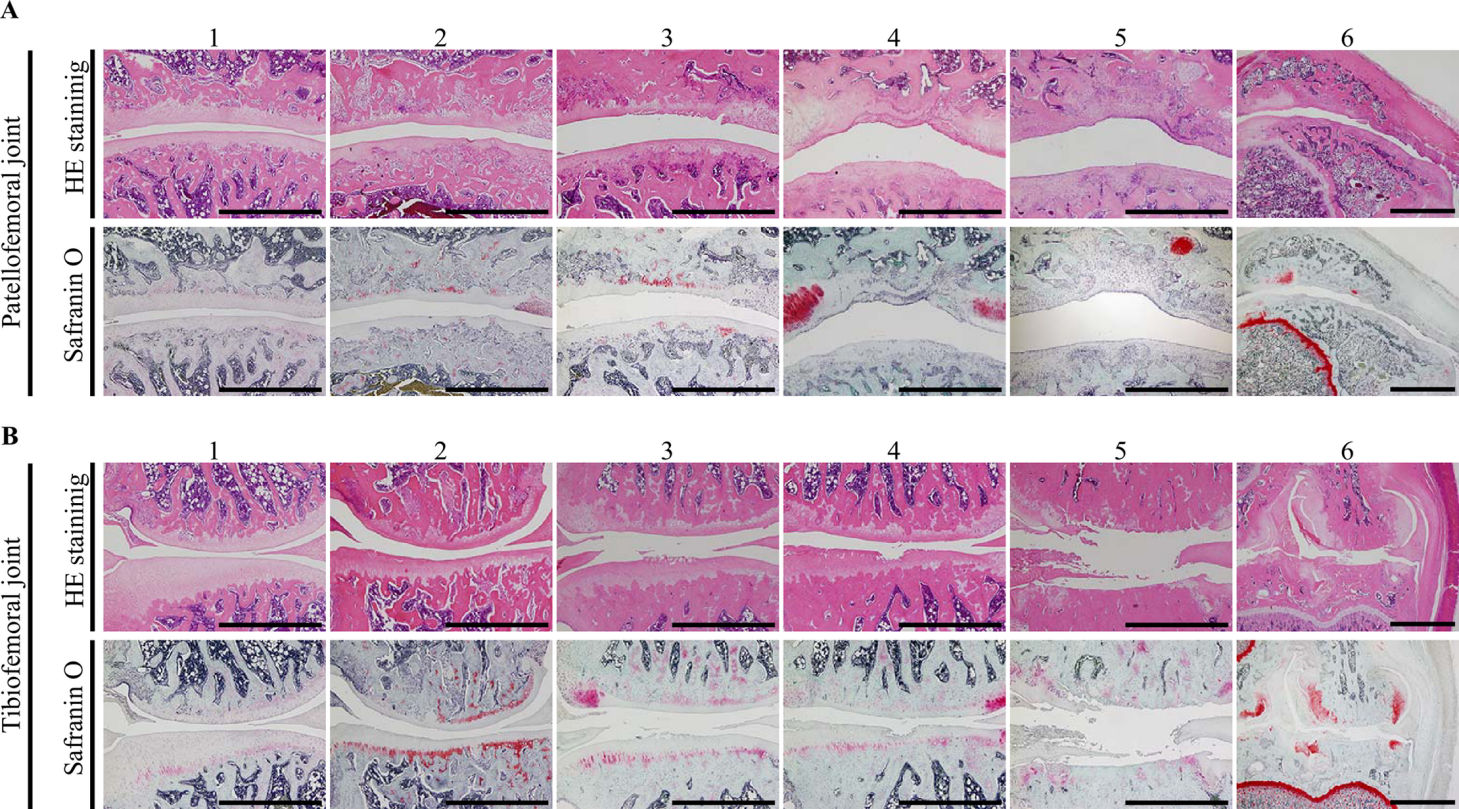
Histopathological features according to the Osteoarthritis Research Society International grade and staining method. Slides from the (A) patellofemoral joint and (B) tibiofemoral joint were stained with hematoxylin and eosin and safranin O. Grade 1, surface intact; grade 2, surface discontinuity; grade 3, vertical fissures; grade 4, erosion; grade 5, denudation and reparative tissue surface present; and grade 6, deformation and osteophyte formation. Scale bar = 1 mm (grades 1–5) and 2 mm (grade 6).

For histological analysis, one representative slice was chosen from each knee and evaluated. To clarify the histopathological changes of OA, we quantitatively evaluated the changes using the OARSI scoring system [27] and the IFP inflammation scoring system [21] (Table 1). The OARSI osteoarthritis cartilage histopathology assessment system (OARSI score) was established by Pritzker et al. [27]; the score ranges from 0 to 24, with higher values indicating more advanced cartilage degeneration (Fig 2A). The IFP inflammation scoring system was established by Udo et al. [21] to evaluate the degrees of synovitis and fibrosis in the IFP. The scoring system comprises a synovitis score and fibrosis score, and the scores range from 0 to 3, with higher values indicating more severe histopathological changes. We evaluated the sections at each region using these scoring systems. The scores were evaluated by a single-blinded and trained independent observer (M.H., pathologist).

**Table 1:** Synovitis score and fibrosis score of the infrapatellar fat pad.

**Points**	**Histological features**
Synovitis at the surface of the IFP	
0	Normal
1	Cellularity is increased; multinucleated cells are present
2	Thickened lining of cells, low (<three-fold thickness of the normal synovial membrane)
3	Thickened lining of cells, high (>three-fold thickness of the normal synovium)
**Points**	**Histological features**
Fibrosis in the body of the IFP	
0	No fibrotic lesion
1	Fibrotic lesion in the IFP is present, low
2	Fibrotic lesion is increased, high
3	IFP is filled with the fibrotic lesion, but fat cells are absent

IFP, infrapatellar fat pad

### Statistical analysis

Statistical analyses were performed using JMP 10 software (SAS Institute, Cary, NC, USA). The sample size was five rats for each subgroup. To evaluate the histopathological changes of the articular cartilage and IFP, we compared the OARSI score and IFP inflammation score. These scores were not normally distributed; therefore, multi-intergroup differences were evaluated using the Steel-Dwass test. Descriptive statistics were calculated as the median and interquartile range for the OARSI score and the IFP inflammation score. A p-value <0.05 was considered statistically significant for all analyses. Exact p-values between groups are presented in the graphs within the figures.

## Results

Within several minutes after the MIA injection, all animals were conscious and started to move. None of the rats showed any signs of knee infection or swelling or died during the experimental period. Therefore, inflammation was macroscopically and microscopically well controlled. Histopathological changes in both the PF and TF joints developed from grade 1 of the OARSI scoring system to grade 6 due to the dose of MIA (Fig 2).

### Histopathological changes in the patellofemoral joints

**Fig 3:**
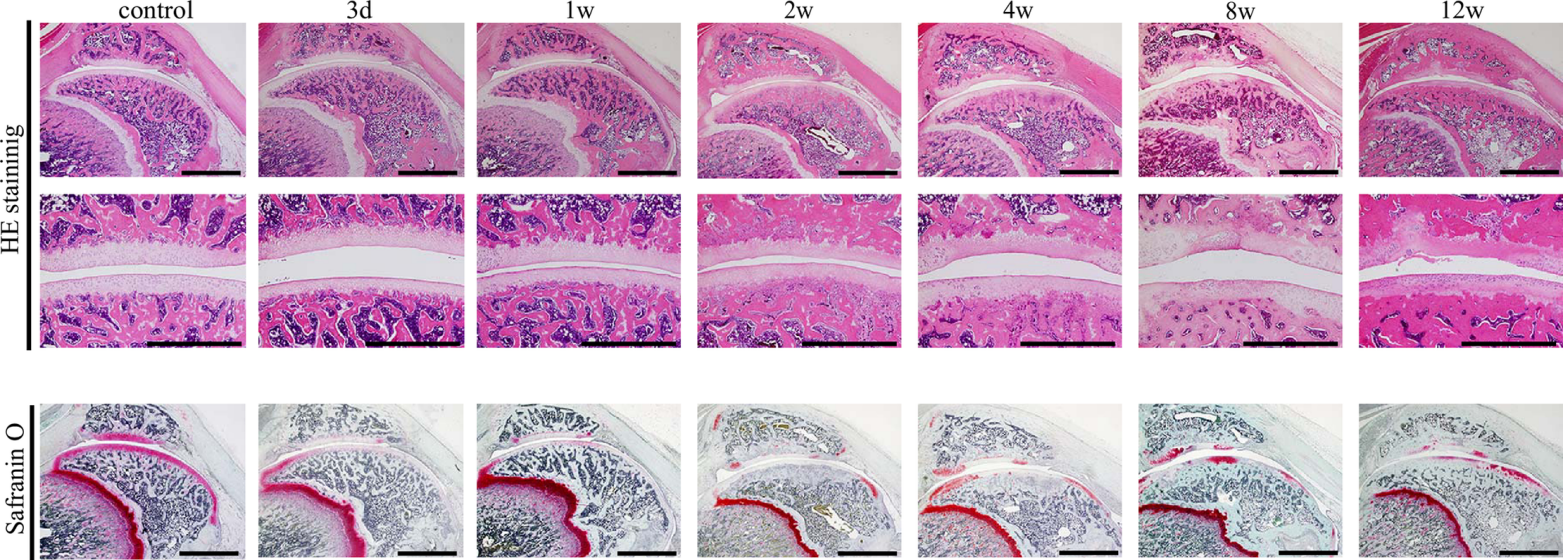
Representative histopathological changes of articular cartilage in the patellofemoral joint induced using 0.2-mg monosodium iodoacetate. The upper and lower sections present an overview of the patellofemoral joint stained with hematoxylin and eosin (HE) and safranin-O fast green. Deformation of the whole joint is not observed. Weak safranin-O staining of the cartilage matrix is observed at 4 weeks. However, staining of the cartilage matrix showed slight regeneration at the margin at 8 and 12 weeks. The middle section shows high magnification images of the contact area of the articular cartilage of the patella and femur stained with HE. Early-stage osteoarthritic histological changes, such as fibrillation and fissuring, are not detected. Scale bar = 2 mm (upper and lower sections) and 1 mm (middle section).

**Fig 4:**
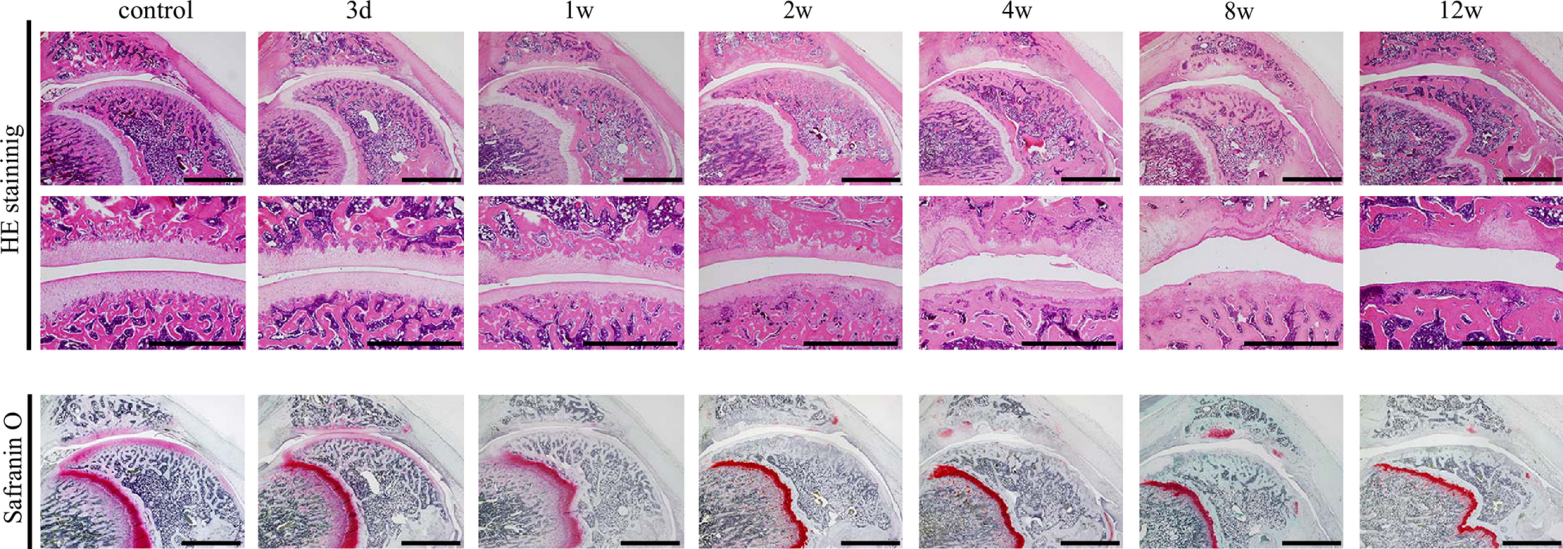
Representative histopathological changes of articular cartilage in the patellofemoral joint induced using 1.0-mg monosodium iodoacetate. The upper and lower sections present the overview of the patellofemoral joint stained with hematoxylin and eosin (HE) and safranin-O fast green. Deformation of the whole joint is observed after 8 weeks. Weak safranin-O staining of the cartilage matrix is observed during the experimental period. The middle section shows high magnification images of the contact area of articular cartilage of the patella and femur stained with HE. Early-stage osteoarthritic histological changes are observed after 4 weeks, and end-stage osteoarthritic changes, such as the eburnation or deformation, are observed after 8 weeks. Scale bar = 2 mm (upper and lower sections) and 1 mm (middle section).

**Fig 5:**
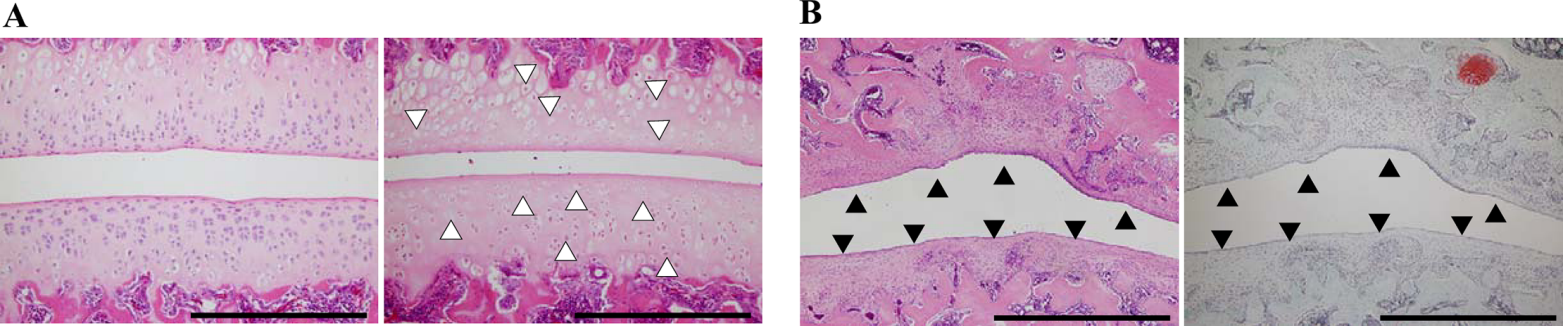
Characteristic histological changes of the articular cartilage in the patellofemoral joint. A: In the left image, normal articular cartilage and chondrocytes of the control group are shown. In the right image, histological changes of the chondrocyte nuclei are observed, including weak hematoxylin and eosin (HE) staining of nuclei, nuclear enlargement, disintegration of nuclei, and differences in nuclear size at the patella and femur (white triangles). B: Replacement of the articular cartilage surface with the membranous structure (black triangles) is shown stained with HE (left image) and safranin-O fast green (right image). Scale bar = 200 μm (A) and 1 mm (B).

For the 0.2-mg (Fig 3) and 1.0-mg groups (Fig 4), weak HE staining of the chondrocyte nuclei, nuclear enlargement, disintegration of nuclei, and differences in nuclear size were detected at the patella and femur from 3 days to 2 weeks after the injection (Fig 5A). In the 0.2-mg group, from 4 to 12 weeks, these histological findings were continuously observed. In the 0.2-mg group, irregularity of the articular cartilage surface was not detected from 3 days to 4 weeks, but slight irregularity was observed after 8 weeks. Conversely, in the 1.0-mg group, early-stage osteoarthritic histopathological changes of fibrillation and fissuring were observed in the patella and femur at 4 weeks. At 8 and 12 weeks, the end-stage osteoarthritic changes of erosion, denudation, and replacement of articular cartilage via fibrous tissue were detected (Fig 5B).

For the 0.2-mg group, weak safranin-O staining of the cartilage matrix was detected at all regions of the patella and femur at 3 days, and at 1 and 2 weeks, no staining of the cartilage was detected at any region of the patella and femur (Fig 3). At 4 weeks, staining of the cartilage matrix showed slight regeneration at the margin, and at 8 and 12 weeks, staining of the cartilage was detected at the margin and the partial contact area of the articular cartilage of the patella and femur. In the 1.0-mg group, weak staining of the cartilage matrix was detected at all regions of the patella and femur at 3 days, while no staining of the cartilage was detected at 1 and 2 weeks (Fig 4). After 4 weeks, staining of the cartilage matrix showed partial regeneration at the articular cartilage margin. However, the staining of the whole matrix was not detected because of the end-stage osteoarthritic changes of erosion, denudation, and replacement of articular cartilage via fibrous tissue.

### Semi-quantitative evaluation using the OARSI score for the patellofemoral joint

**Fig 6:**
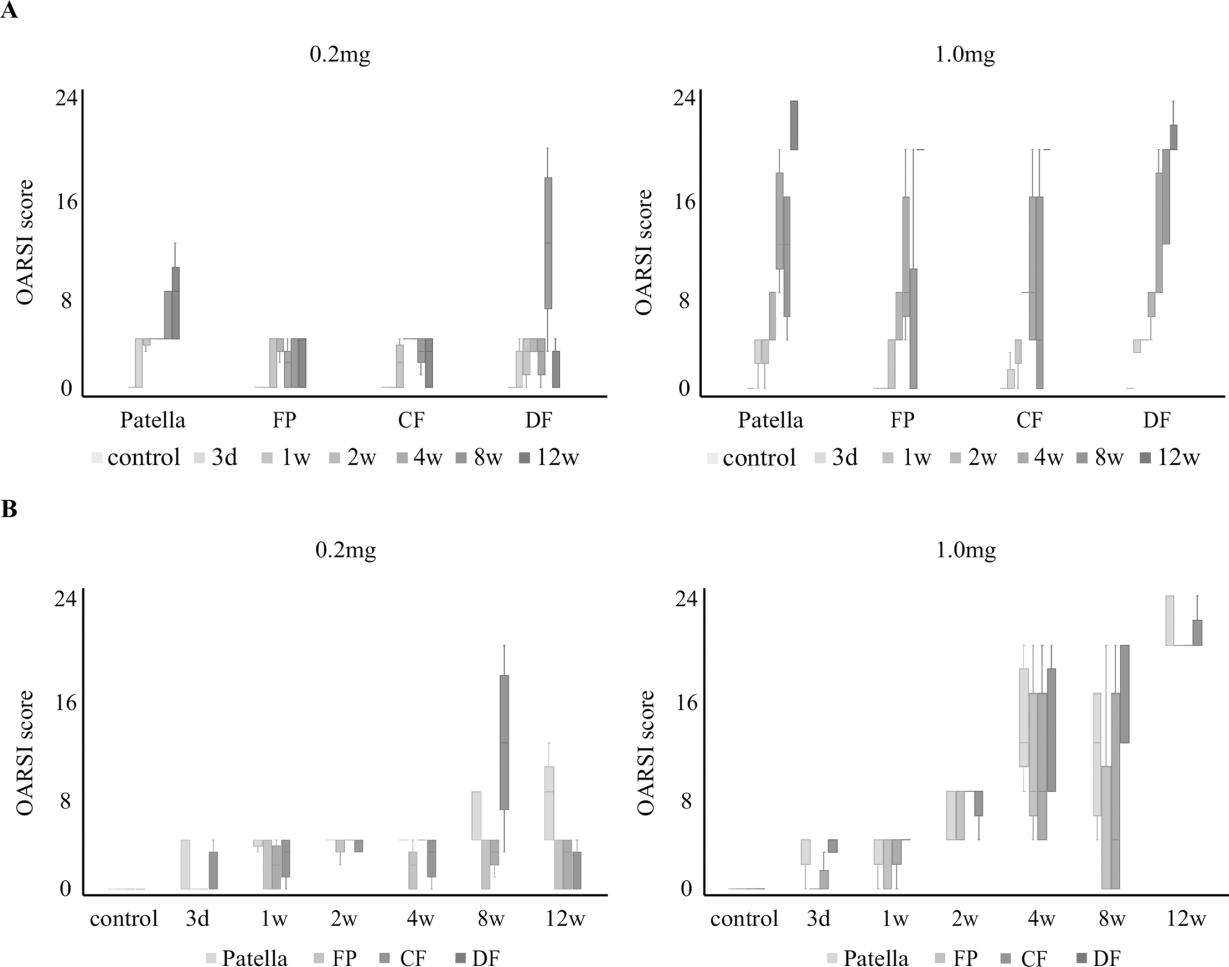
Articular cartilage degeneration in the regions of the patellofemoral joint using the OARSI scoring system. A: Changes in the OARSI scores of each region according to time. B: Differences in the OARSI scores between regions. Boxplots display median values and interquartile ranges. Articular cartilage region: proximal femur (FP), central femur (CF), distal femur (DF), and patella. There is no statistically significant difference in any of the results. OARSI, Osteoarthritis Research Society International.

Results of the OARSI score for the PF joint by each evaluation time are shown in Fig 6A. In the 0.2-mg group, scores of the patella tended to increase over time, but there were no significant differences. Scores of the femur remained low. In the 1.0-mg group, the scores of all regions tended to increase over time, but there were no significant differences between the evaluation times.

Results of the OARSI score for the PF joint separated by region are shown in Fig 6B. In the 0.2-mg group, the score of each region from 3 days to 4 weeks remained low, and the scores at 8 and 12 weeks were increased by region. However, there were no significant differences in the scores between the regions of the PF joint. Additionally, in the 1.0-mg group, there were no significant differences in the scores between the regions of the PF joint.

### The histopathological changes and OARSI score in the tibiofemoral joint

**Fig 7:**
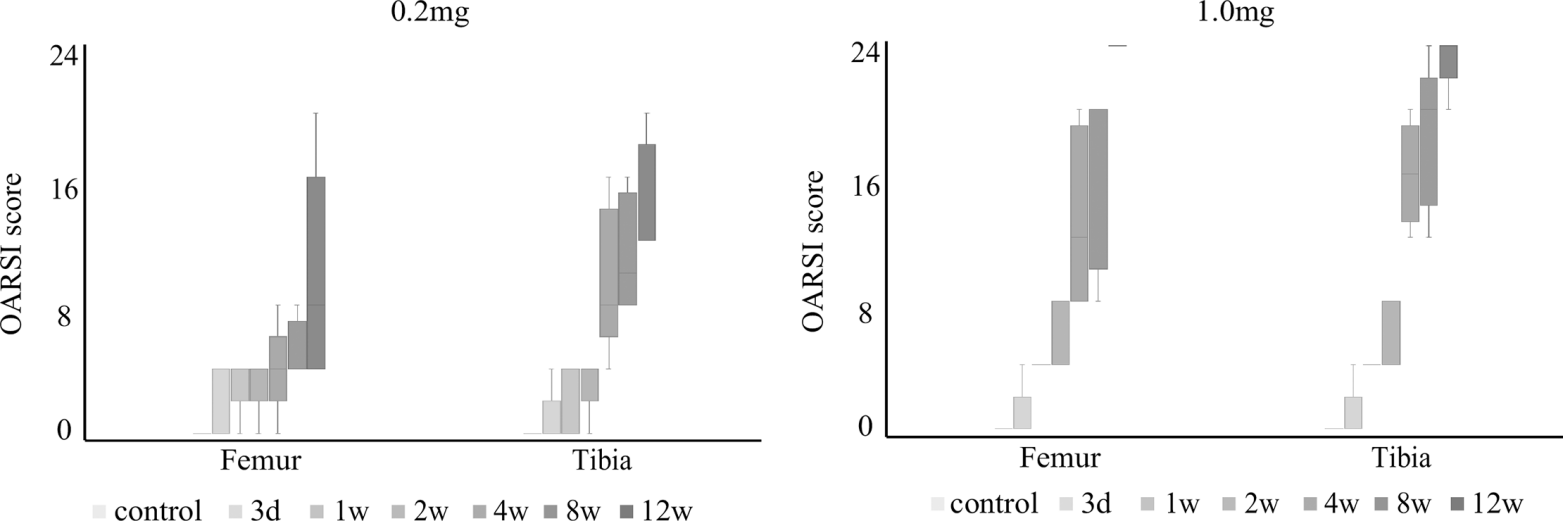
Articular cartilage degeneration in the tibiofemoral joint using the OARSI scoring system. Changes in the OARSI scores over time in the 0.2-mg and 1.0-mg groups. Boxplots display median values and interquartile ranges. In the 0.2-mg and 1.0-mg groups, the scores of the femur and tibia tended to increase over time, but there were no significant differences. OARSI, Osteoarthritis Research Society International.

The histopathological features according to the OARSI grade for the TF joint are shown in Fig 2B, and the results of the OARSI score for the TF joint are shown in Fig 7. The scores tended to increase over time, but there were no significant differences between the evaluation times for either region and both dose groups.

For the 0.2-mg group, weak HE staining of chondrocyte nuclei, nuclear enlargement, disintegration of nuclei, and differences in nuclear size were detected at the femur and tibia from 3 days to 2 weeks. At 4 weeks, fibrillation was observed, and at 8 weeks, fissuring was observed at the central area of the tibia. At 12 weeks, erosion and denudation of the articular cartilage were detected in the femur and tibia. Conversely, in the 1.0-mg group, histopathological changes developed earlier than they did in the 0.2-mg group. At 1 week, no staining of the cartilage matrix was observed, while at 2 weeks, fibrillation was detected. At 4 weeks, fissuring and erosion were observed, and at 8 weeks, denudation of the articular cartilage was detected at the contact area of the femur and tibia. At 12 weeks, joint deformation at the whole joint and osteophyte formation at the articular cartilage margin were detected.

In the 0.2-mg group, weak safranin-O staining of the cartilage matrix was detected at all areas of the femur and tibia at 3 days, and at 1 and 2 weeks, no staining of the cartilage was detected at any area of the femur and tibia. At 4 weeks, there was still no staining of the cartilage matrix at the contact area; however, staining of the cartilage matrix regenerated at the articular cartilage margin. At 8 and 12 weeks, the findings at 4 weeks were continuously detected. In the 1.0-mg group, weak staining of the cartilage matrix was detected at all areas of the femur and tibia at 3 days, whereas at 1 week, no staining of the cartilage was detected. After 4 weeks, histopathological findings in the TF joint were similar to those at 4 weeks in the 0.2-mg group.

### Semi-quantitative evaluation using the infrapatellar fat pad inflammation score

**Fig 8:**
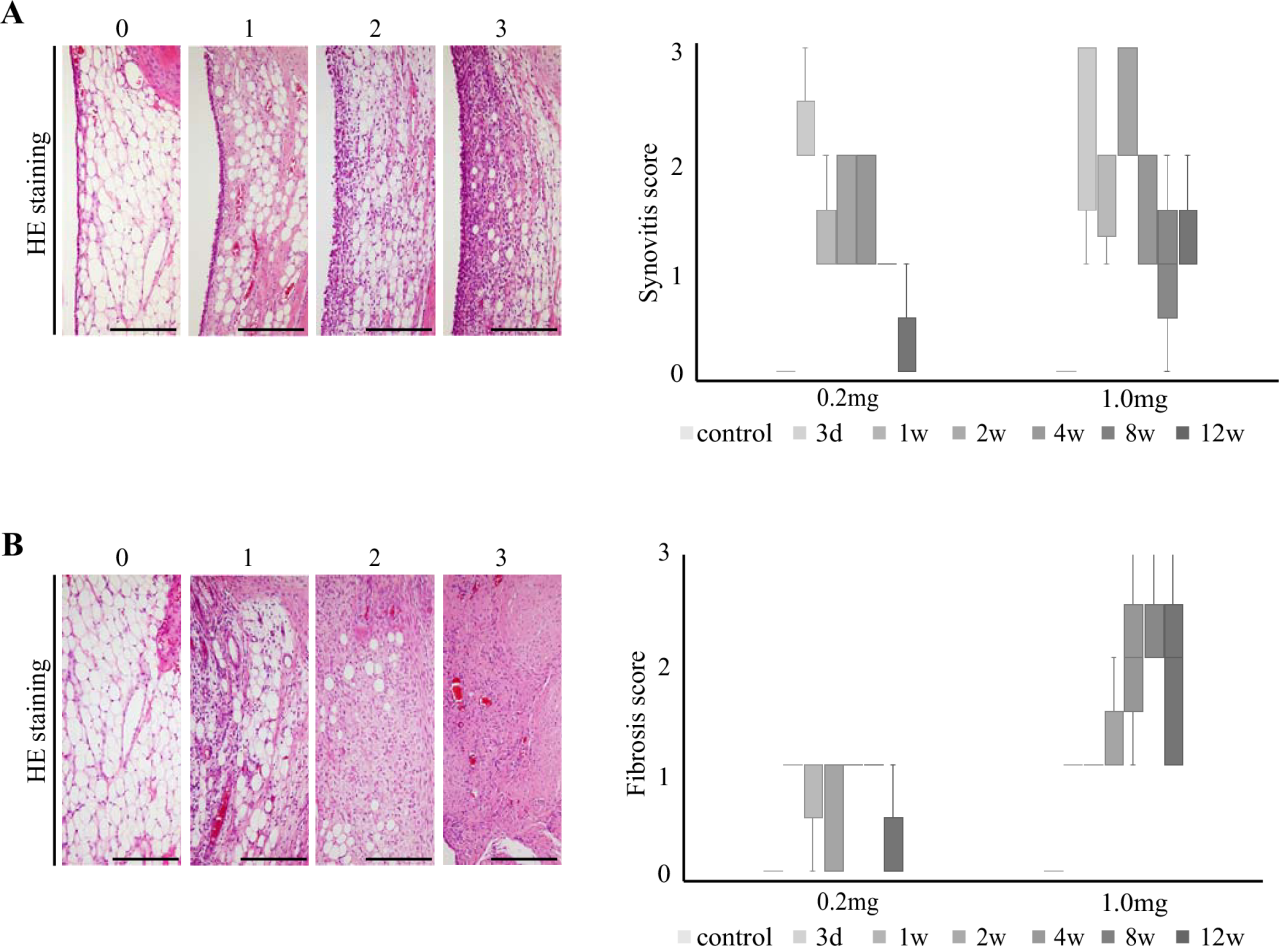
Representative histopathological features and changes in infrapatellar fat pad inflammation scores over time. Histological features were examined according to the infrapatellar fat pad inflammation scores for the (A) surface and (B) body of the infrapatellar fat pad. Changes in the (A) synovitis and (B) fibrosis scores of the infrapatellar fat pad were analyzed by group over time. The synovitis score is deceased over time. The fibrosis score in the 0.2-mg group remains low, but the score in the 1.0-mg group is increased over time. Scale bar = 200 μm.

The results of the synovitis and fibrosis scores for the IFP are shown in Fig 8. The synovitis score of the IFP was high during the early phase in both groups, at which point the score tended to decrease over time (Fig 8A). The fibrosis score of the IFP for the 1.0-mg group tended to increase with time, whereas that of the 0.2-mg group remained low (Fig 8B). In the synovitis score and fibrosis score, there were no statistically significant differences in any of the results.

## Discussion

There have been some reports of the histopathological changes of PFOA. For example, Mori et al. used arthroscopy to evaluate the histological changes of patellar chondropathy in adolescents. The researchers found localized fibrillation, fissuring, swelling, and proliferation of undifferentiated interlobular mesenchymal cells [28]. Meachim et al. reported that fibrillation or full-thickness cartilage defects in the PF joint were detected during autopsy [29]. Using arthroscopy, Hjelle et al. reported that softening, cartilage swelling, and focal lesions were detected in humans [30]. According to a basic study using animals, an OA model for the PF joint has not been established; however, some researchers have reported that anterior cruciate ligament transection (ACLT) induces histopathological changes in the PF joint. Clark et al. used felines to inspect the histopathological changes in the articular cartilage of the PF joint after ACLT, and showed some erosion at 4 months and loss of proteoglycan staining at 5 years [31]. Chang et al. investigated the histopathological changes after ACLT in rabbits, and reported that OA changes were detected in the superficial and middle zones of the articular cartilage at 4 weeks postoperatively [32]. Moreover, Tsai et al. investigated the histological changes in the PF joint cartilage that resulted in subchondral marrow edema at 16 weeks and matrix degeneration with irregular surfaces at 32 weeks after ACLT in rats [33]. Egloff et al. investigated the effect of muscle weakness using surgical denervation and intramuscular drug injection on the progression of OA, and exhibited an increase in the OARSI score of the PF joint at 3 months postoperatively [34]. Chia et al. used a newly designed surgical model to induce OA in mice and stated that the PF joint showed evidence of mild OA at 8 weeks and further degeneration at 12 weeks after surgery [35]. Though these studies were able to report these changes in PFOA, the studies reported findings that had problems with temporal, not sequential, histopathological evaluation and the reproduction of only mild OA or OA during the early stage.

To the best of our knowledge, this is the first study to focus on the sequential histopathological changes of OA in the PF joint. As a result, representative histopathological changes of OA were detected, such as fibrillation, fissuring, erosion, denudation, and osteophyte formation, and we succeeded in reproducing the time course of OA from the early stage to the end stage. In addition, Mori et al. observed the proliferation of undifferentiated interlobular mesenchymal cells on the PF joint surface in humans. In the present study, articular cartilage surface invasion of membranous structures, similar to those of the previous study, was observed, and it was histopathologically remarkable because our study reproduced the histological changes of human OA in the PF joint of rats. In previous studies using animals, it was necessary to induce the histological changes of OA for a long period, such as months to years [31–34]. In the present study, it was worthwhile to induce osteoarthritic changes during the end stage for 12 weeks after the MIA injection.

Many researchers have described the histopathological changes of the articular cartilage in the TF joint after MIA injection, such as degeneration of the articular cartilage and subchondral bone and necrosis of the chondrocyte [18, 19, 23, 36–40]. However, these previous studies that induced OA using MIA injections focused only on the TF joint. In the present study, histopathological changes in the PF joint were similar to those in the TF joint. This finding suggests that histopathological changes of PFOA are similar to those of TFOA. However, there were three differences between PFOA and TFOA in the present study. First, the speed of progression of OA in the PF joint was slower than that in the TF joint. Second, distribution of the OARSI score in the PF joint was wider than that in the TF joint. Third, the presence of the membranous structure at the articular cartilage surface was only found in the PF joint. Determining factors for these three differences are unclear; however, Clark considered three possible influential factors on the disparate progression of PFOA: 1) load duration; 2) histological, material, and compositional properties; and 3) chondrocyte anabolic or catabolic metabolism [41]. Kobayashi et al. reported that the PF and TF joints exhibit different structural, pathomechanical, and clinical characteristics [2]. These factors may have induced the histopathological differences in the present study.

The amount of MIA injection was an important factor for the progression of OA. In previous studies, it was reported that the degree of histopathological changes was dose-dependent [19, 37]. Udo et al. regarded 0.2 mg of MIA as the representative low dose in the MIA model and 1.0 mg of MIA as the representative high dose in the MIA model. Furthermore, the researchers reported that MIA-induced arthritis progressed in a dose-dependent and time-dependent manner [21]. Other researchers have reported that in rats, 1.0 mg of MIA was the maximal effective dose for inducing OA [16]. In the present study, the progression of OA in both the PF and TF joints in the 1.0-mg group was faster than that in the 0.2-mg group, and the results of the present study support those of previous studies.

A quantitative evaluation of synovitis and fibrosis in the IFP was performed using a scoring system in the present study [21]. Although OA is considered a non-inflammatory condition, it is widely accepted that synovial inflammation is a feature of OA [42, 43]. Udo et al. described the changes of the sum of the inflammation score and the synovitis and fibrosis scores over time; however, it was unclear how the synovitis and fibrosis scores changed. Therefore, we investigated the changes in these scores independently over time. We revealed that both scores decreased in the 0.2-mg group, and that the synovitis score decreased in the 1.0 mg group, while the fibrosis score increased with time. These results may suggest that mild and severe synovitis and mild fibrosis in the IFP are reversible, whereas severe fibrosis is irreversibly induced using MIA.

Three limitations of this study were identified. First, there was no significant difference in the OARSI score and IFP score, likely because of the small number of specimens assessed. However, the quantitative results showed some clear trends, and we believe the results in the present study are useful and valuable for induction of a PFOA model. Second, investigation of the articular cartilage in the PF joint was only performed in the sagittal plane. With regard to the study design, a histopathological analysis of the articular cartilage in an additional horizontal plane is recommended. Regardless, the present study’s results adequately contributed to the elucidation of the pathological mechanism and understanding of PFOA. Third, we used the chemical drug MIA to induce OA. Chemical models are reported to have a unique pathophysiology that has no association with post-traumatic OA [15, 20, 24, 44]; however, some researchers have reported histological and morphological articular alterations that are similar to the changes in human OA [16–19, 45]. Therefore, this method may be useful for determining whether osteoarthritic changes originate from cartilage or subchondral bone alterations [16].

In conclusion, the injection of MIA into the rat’s knee induced histopathological changes (fibrillation, fissuring, erosion, denudation, and osteophyte formation) in the PF joint. These histopathological changes were similar to those of TFOA, but not in the presence of the membranous structure. This result suggests that it is possible to create a PFOA model by the administration of MIA with high reproducibility and accuracy, and a shorter experimental period. Additionally, this model may be useful and valuable for studying pain-related behavior and the therapeutic efficacy of potential agents for PFOA treatment.

## Supporting information

S1 File NC3Rs ARRIVE guidelines checklist. (PDF)
